# A Deletion of the Nuclear Localization Signal Domain in the Fus Protein Induces Stable Post-stress Cytoplasmic Inclusions in SH-SY5Y Cells

**DOI:** 10.3389/fnins.2021.759659

**Published:** 2021-12-23

**Authors:** Antonietta Notaro, Antonella Messina, Vincenzo La Bella

**Affiliations:** ALS Clinical Research Center and Laboratory of Neurochemistry, Department of Biomedicine, Neuroscience and Advances Diagnostics, University of Palermo, Palermo, Italy

**Keywords:** amyotrophic lateral sclerosis, Fused-in-Sarcoma protein, nuclear localization signal (NLS), stress granules (SG), cytoplasmic inclusions

## Abstract

Mutations in Fused-in-Sarcoma (FUS) gene involving the nuclear localization signal (NLS) domain lead to juvenile-onset Amyotrophic Lateral Sclerosis (ALS). The mutant protein mislocalizes to the cytoplasm, incorporating it into Stress Granules (SG). Whether SGs are the first step to the formation of stable FUS-containing aggregates is still unclear. In this work, we used acute and chronic stress paradigms to study the SG dynamics in a human SH-SY5Y neuroblastoma cell line carrying a deletion of the NLS domain of the FUS protein (homozygous: ΔNLS^–/–^; heterozygous: ΔNLS^+/–^). Wild-type (WT) cells served as controls. We evaluated the subcellular localization of the mutant protein through immunoblot and immunofluorescence, in basal conditions and after acute stress and chronic stress with sodium arsenite (NaAsO_2_). Cells were monitored for up to 24 h after rescue. FUS was expressed in both nucleus and cytoplasm in the ΔNLS^+/–^ cells, whereas it was primarily cytoplasmic in the ΔNLS^–/–^. Acute NaAsO_2_ exposure induced SGs: at rescue,>90% of ΔNLS cells showed abundant FUS-containing if compared to less than 5% of the WT cells. The proportion of FUS-positive SGs remained 15–20% at 24 h in mutant cells. Cycloheximide did not abolish the long-lasting SGs in mutant cells. Chronic exposure to NaAsO_2_ did not induce significant SGs formation. A wealth of research has demonstrated that ALS-associated FUS mutations at the C-terminus facilitate the incorporation of the mutant protein into SGs. We have shown here that mutant FUS-containing SGs tend to fail to dissolve after stress, facilitating a liquid-to-solid phase transition. The FUS-containing inclusions seen in the dying motor neurons might therefore directly derive from SGs. This might represent an attractive target for future innovative therapies.

## Introduction

Amyotrophic lateral sclerosis (ALS) is a severe neurodegenerative disorder characterized by the progressive and selective loss of the upper and lower motor neurons (MN) in the brain and spinal cord. A genetic cause has been identified in most familial cases, and several sporadic cases have been associated with ALS-related genes (Brown and Al-Chalabi, 2017; Gibson et al., 2017; Srinivasan and Rajasekaran, 2020). Gene mutations play therefore a pivotal role in the etiopathogenesis of the disease.

Mutations in genes coding for RNA/DNA-binding proteins (RBPs), such as TARDBP, located on chromosome 1 p36.22, and FUS, located on chromosome 16 p11.2, are associated with ALS and are implicated in the pathogenesis of the disease (Neumann et al., 2006; Van Deerlin et al., 2008; Kwiatkowski et al., 2009; Vance et al., 2009; Ederle and Dormann, 2017).

Both TDP-43 and FUS proteins are predominantly located in the nucleus. They usually shuttle between the nucleus and cytoplasm, regulating the RNA transcription and splicing, mRNA metabolism and transport (Ayala et al., 2008; Dormann et al., 2010; Lagier-Tourenne et al., 2010; Birsa et al., 2020). Interestingly, post mortem analysis of sporadic and familial ALS patients showed abnormal aggregation of these proteins in the brain (Arai et al., 2006; Deng et al., 2010; Hewitt et al., 2010). Whether the FUS-containing cytoplasmic aggregates play a pivotal role in ALS pathogenesis is still unknown.

Since the first descriptions in familial ALS, several FUS/TLS gene mutations have been identified (Kwiatkowski et al., 2009; Nolan et al., 2016; Yousefian-Jazi et al., 2020). Most mutations are located in the exon 15 of the gene, and they are often associated with a young-onset ALS with relatively rapid progression (Chiò et al., 2009; Conte et al., 2012; Lagier-Tourenne et al., 2012; Kuang et al., 2017). Exon 15 encodes for the C-terminal region of the protein, containing the nuclear localization signal (NLS) domain, which is formed by an arginine-glycine-rich region (RGG3) followed by a proline-tyrosine (PY) consensus sequence (Dormann et al., 2012; Hofweber et al., 2018). Several proteins (i.e., Transportin-1 (TNPO-1), Transportin-3, importin β, importin 7 and importin β/7 dimer) can chaperone and import FUS to the nucleus through the RGG (i.e., RGG1, RGG2, RGG3) and the PY motifs (Baade et al., 2021).

TNPO-1 appears to be the most efficient importin for FUS thanks to its binding with the PY motif of the NLS. Mutations in NLS, particularly the mutation P525L, prevent an efficient interaction with the nuclear import receptor TNPO-1. Consequently, the protein heavily mislocalizes to the cytoplasm in symptomatic and asymptomatic carriers (Dormann et al., 2010; Lo Bello et al., 2017). Noteworthy, in fibroblasts of FUS P525L mutation carriers, the mutant protein appears to be expressed only in the cytoplasm in a high proportion of the cells, suggested to be a putative phenoconversion marker from an asymptomatic to symptomatic condition (Caputo et al., 2020).

FUS is a crucial regulator of RNA processing, and it plays important roles both in the nucleus and in the cytoplasm (Dormann, 2016; Ederle and Dormann, 2017; Birsa et al., 2020). The nuclear functions of FUS involve transcription, pre-mRNA splicing and processing of non-coding RNAs (Ederle and Dormann, 2017). Furthermore, FUS plays a role in mRNA trafficking and stability in the cytoplasm and possibly also in mRNA translation (Colombrita et al., 2012; Ratti and Buratti, 2016; Ederle and Dormann, 2017; Birsa et al., 2020). Finally, as mRNAs along with RNA-binding proteins (RNPs) form transport granules that travel along axons and dendrites, FUS enters in those granules and may play an active role in mRNA trafficking toward the neuronal terminals (Belly et al., 2005; Doyle and Kiebler, 2011; Zhang et al., 2012).

FUS protein, either wild-type or mutant, can be sequestered into the stress granules (SG) (Dormann et al., 2010; Meyerowitz et al., 2011; Li et al., 2013; Lo Bello et al., 2017; Fernandes et al., 2018). SGs are cytoplasmic ribonucleoprotein complexes that rapidly self-associate to limit the translation of mRNAs when cells are exposed to potentially dangerous stressors (Anderson and Kedersha, 2008; Riggs et al., 2020). They are membrane-less transient structures that rapidly disassemble upon the removal of stress.

The role of SGs in ALS pathogenesis is still controversial. Still, it is suggested, though not demonstrated, that FUS- or TDP-43-containing SG might represent a first step to the pathological protein aggregation leading to motor neurons degeneration (Buratti and Barrale, 2010; Dormann et al., 2010; Li et al., 2013; Fernandes et al., 2018; Ratti et al., 2020).

We have recently demonstrated that mutant FUS P525L protein is mislocalized to the cytoplasm of fibroblasts of asymptomatic carriers and incorporated into SG after cellular stress (Lo Bello et al., 2017). The mutant FUS P525L-containing SGs appear to persist longer than those containing the wild-type (WT) protein (i.e., from patients with sporadic ALS and healthy controls), suggesting a pre-aggregative state.

Mutant FUS accumulating in the cytoplasm and forming intracellular inclusions is undoubtedly involved in motor neuron degeneration (Dormann, 2016). However, whether the mutant FUS P525L-containing cytoplasmic aggregates come from persistent SGs, which by sequestering mRNAs and other RNPs lead to irreversible alteration of the motor neuron metabolism and activity, is still a matter of debate (Dormann et al., 2010; Li et al., 2013; Fernandes et al., 2018).

In the present work, we used the human SH-SY5Y neuroblastoma cells permanently transfected with FUS bearing a deletion of the C-terminus containing the nuclear localization signal domain (NLS) to study the effect of either acute or chronic stress on SGs formation and persistence. We found that the NLS-lacking FUS protein formed in the SH-SY5Y neuroblastoma cells stably and long-lasting SGs, representing the first cellular model for the persistent cytoplasmic FUS-containing inclusions seen in the disease.

## Experimental Procedures

The experiments were carried out following the World Medical Association Declaration of Helsinki. The Ethics Committee of the University of Palermo approved the study protocol (Palermo 1, n°4/2019).

### Cell Culture and Maintenance

We used undifferentiated SH-SY5Y human neuroblastoma cells, which express a deletion of the human FUS/TLS gene corresponding to the C-terminus of the protein. The deleted region encodes for the last fourteen amino acids (i.e., from serine 513 to tyrosine 526, Supplementary Figure 1), partially corresponding to the nuclear localization signal domain (NLS), known to be implicated in the nuclear transport of the protein (Dormann et al., 2010).

Mutant cells, either homozygous (ΔNLS^–/–^) or heterozygous (ΔNLS^+/–^), were produced using the CRISPR/Cas9 editing procedure according to a published protocol (Cong et al., 2013) and were a gift of Tatiana A. Shelkovnikova from the Biomedicine Division, School of Biosciences, Cardiff University, Cardiff, United Kingdom (An et al., 2019). Wild-type (WT.) SH-SY5Y were used as controls.

Cells were grown in a 1:1 mixture of Dulbecco’s Modified Eagle’s Medium (DMEM) and F12 Medium supplemented with 10% fetal bovine serum (FBS), 100 U/mL penicillin, 100 μg/mL streptomycin and 2 mM L-glutamine (all from Euroclone, Pero, Italy) in a humidified atmosphere containing 5% CO_2_. Cells were maintained in culture through passages on flask, and the medium was changed every 2–3 days.

All experiments were performed with cells at their 3rd–4th passage on flask.

### Sodium Dodecylsuphates-PAGE and Immunoblot

Protein extracts were mixed with Sodium Dodecylsuphate (SDS) sample buffer and heated at 95° C for 5 min. Proteins were separated in a 10% SDS Polyacrylamide gel by electrophoresis, and then immunoblot was performed. Prestained molecular weight markers were used in all experiments.

Blots were blocked with 10% powdered milk in PBS-Tween 0.5% and then incubated overnight at 4°C with a polyclonal FUS antibody (Proteintech Group Inc., Chicago, IL, United States) at 1:1,500 dilution. Secondary Horse Radish Peroxidase-conjugated donkey anti-rabbit antibody (Millipore, Billerica, MA, United States) was used at a dilution of 1:5,000 in 1% milk/PBS-Tween 0.5%. Filters were washed with PBS-Tween 0.5% and protein bands visualized with the SuperSignal^®^ West Pico Chemiluminescent Substrate (Pierce Biotechnology, Rockford, IL, United States). The detection of chemiluminescence was performed by the ChemiDoc-It Imaging System (UVP, Cambridge, United Kingdom).

In some experiments, filters were washed and stripped out of the bound antibodies (La Bella et al., 2000). After blocking, membranes were reused to detect the nuclear fraction marker Sp1 using a polyclonal anti-Sp1 antibody (Santa Cruz Biotechnology Inc., Dallas, TX, United States) at 1:200 dilution.

### Preparation of Soluble and Insoluble Cellular Fractions

Cells were scraped on ice with lysis buffer (PBS containing 0.1% Triton X100 and protease inhibitors) to obtain soluble and insoluble fractions and left on ice for 30 min with periodic gentle vortexing. The lysates were then centrifuged at 17,000 g for 20 min at 4°C. The supernatant and the pellet were recovered as soluble and insoluble fractions, respectively (Shelkovnikova et al., 2014). The protein content in each fraction was quantified with BCA™ method (Pierce Biotechnology, Rockford, IL, United States). Equal amounts of proteins (i.e., 20 μg) were loaded on a 10% SDS-electrophoresis gel, and immunoblot was performed using a polyclonal anti-FUS antibody to detect the RNA-binding protein in each fraction.

### Immunofluorescence

Cells were cultured on glass coverslips, pre-coated with 0.2% gelatin, for 24 h before being fixed with 4% paraformaldehyde. As previously described, fixed cells were processed for immunofluorescence (La Bella et al., 1998).

For FUS detection, we used a polyclonal anti-FUS antibody at 1:1,000 dilution. For double staining experiments and SGs detection, we used a polyclonal hFUS antibody and the monoclonal TIA-R antibody (BD Transduction Laboratories™, San José, CA, United States), the latter as a marker of SGs, both at 1:500 dilution. As a further control for SGs detection, we also used the G3BP1 antibody (BD Transduction Laboratories™, San José, CA, United States) at 1:750 dilution. Cells were incubated with the primary antibodies overnight at 4°C.

After washing with PBS containing 0.1% Triton X-100, cells were incubated with a secondary Cy3-conjugated donkey anti-rabbit antibody (Chemicon International Inc., Temecula, CA, United States) at 1:1,500 dilution for FUS detection. The secondary antibody used for TIA-R detection was a FITC-conjugated donkey anti-mouse antibody at 1:1,500 dilution (Chemicon International Inc., Temecula, CA, United States).

Cells co-stained with FUS and TIA-R were analyzed with a Zeiss LSM 5 EXCITER laser confocal microscope with an argon-ion laser set at 548 nm for Cy3 excitation or 495 nm for FITC excitation.

### Subcellular Fused-in-Sarcoma Expression

FUS is a nuclear RNA-binding protein that heavily mislocalizes to the cytoplasm when it carries a mutation in the NLS domain (Dormann et al., 2010; Lo Bello et al., 2017).

To study the subcellular FUS expression, WT, FUS ΔNLS^–/–^ and FUS ΔNLS^+/–^ SY-SH5Y cells were immunostained with anti-FUS Ab and analyzed using an IX70 Olympus Fluorescent Microscope with the fluorescence lamp set at 548 nm for Cy3 excitation.

We counted the number of cells showing the FUS staining, categorized as follows: (i) cells with an exclusive nuclear FUS staining, the cytoplasmic staining being faint or absent; (ii) cells with uniform nuclear and cytoplasmic staining; (iii) cells with exclusive cytoplasmic staining, the nuclear staining being faint or absent (Caputo et al., 2020).

Analysis was made by combining the data of three independent experiments. Two of us blindly and independently recorded each staining pattern in the cells present in two microscopic fields upward and two other fields downward from the pre-marked center of the coverslip. We thus analyzed a median of 350 ± 35 cells in the four fields in each coverslip. The inter-rater agreement for each pattern of staining was consistently above 90%. Each experiment was carried out in duplicate.

### Subcellular Fractionation

We performed subcellular fractionation of the undifferentiated SH-SY5Y cells to separate the two major cell compartments, i.e., nuclei, and cytoplasm, as described (La Bella et al., 2000). Briefly, cells at 70–80% confluence were resuspended in RBS100 buffer (10 mM Tris-HCl, pH 7.4, 100 mM NaCl) containing 40 μg/mL digitonin, 1 mM PMSF and a cocktail of protease inhibitors (Merck KGaA, Darmstadt, Germany) and incubated at 4°C for 10 min.

Cells were disrupted by passages through a syringe, and the suspension was centrifuged at 900 g for 10 min at 4°C to obtain a supernatant (cytoplasmic fraction) and a pellet (nuclei, Supplementary Figure 2). Both nuclei and cytoplasmic protein content were quantified with a BCA™ protein reagent kit; an equal amount of proteins (i.e., 10 μg) were separated through SDS-PAGE and then processed for immunoblot with the anti-FUS antibody.

### Stress Paradigms and Treatment

For the stress experiments, cells were seeded at 70–80% confluence for western blotting and 40–50% confluence for immunofluorescence, cell counting and stress granules (SG) analysis. After exposure to the stress-inducing chemical, cells were rescued by washes with PBS and replacing the medium containing the stress agent with a fresh one. All analyses were performed at selected times, starting at T0 (i.e., immediately after wash-out) and then at T1.5, T3.0, T6.0, T9.0, and T24 h after rescue. Cells unexposed to the stress chemical were used as controls and analyzed at the same time intervals.

#### Acute Stress

SH-SY5Y cells stably expressing FUS ΔNLS^–/–^ or FUS ΔNLS^+/–^ and the WT cells were incubated with 0.5 mM sodium (meta) arsenite (NaAsO_2_) for 1 h to acutely induce SG formation (Supplementary Figure 3A; Khalfallah et al., 2018).

Cycloheximide (CHX) was used at the concentration of 50 μM in co-treatment with NaAsO_2_ to prevent SG formation.

#### Chronic Cellular Stress

For long-lasting stress, FUS ΔNLS^–/–^, FUS ΔNLS^+/–^ and WT cells were exposed at different concentrations of NaAsO_2_ (i.e., from 0.5 to 50 μM) for 24 h (Supplementary Figure 3B; Khalfallah et al., 2018; Ratti et al., 2020). We found the 10 μM was the optimal concentration of NaAsO_2_ that did not cause cell death, and it was used in all subsequent experiments.

In acute and chronic stress experiments, the untreated ΔNLS^–/–^, ΔNLS^+/–^, and WT cells were used as controls. At different times after rescue, cells were processed for cell counting and immunocytochemistry experiments.

### Cell Viability and Proliferation Studies

The stress agent NaAsO_2_ induces cell degeneration and death, mainly through an apoptotic process (Keim et al., 2012). In our model, cell viability was good in the WT after acute stress being ∼85–90% and decreased to ∼70–75% in the mutant ones (Supplementary Figure 4). The cell viability was higher than 85% in all cell types after chronic stress (*data not shown*).

Cell proliferation after NaAsO_2_ was assessed by direct cell counting. Before plating the cells in 4-well plates, three adjacent 1 mm^2^ microscopic fields in each well were marked. Cells were seeded at 5,000 cells/well, left overnight in the incubator and then exposed to the stress chemical at the given times before being visually counted with the phase-contrast light IX70 Olympus microscope.

### Time Course of the Number of Cells Containing Stress Granules

After induction of either acute stress with 0.5 mM NaAsO_2_ or chronic stress with 10 μM NaAsO_2_, we analyzed the cellular expression and morphology of the SGs through a co-staining with anti-FUS and anti-TIA-R antibodies.

Each glass coverslip was coded, and the proportion of FUS-positive and TIA-R-positive cells containing SGs was independently recorded by two of us under the IX70 Olympus Fluorescent Microscope. At least 200 consecutive cells were analyzed in each coverslip. The analysis was made at different times after rescue (i.e., T0, T1.5, T3.0, T6.0, T9.0, and T24 h).

We took pictures of different microscopic frames with an Olympus C-5060 Camedia digital camera. Images were then transferred to a computer for analysis. Inter-rater agreement was above 88%; hence, data from the two independent counts were pooled together.

Analysis was made with the combined data of three independent experiments.

### Data Analysis and Statistics

All experiments carried out after acute or chronic stress induction (i.e., cell proliferation, the subcellular FUS localization, the time-course analysis of the number of SGs-containing cells) were performed by plating approximately 5,000 cells *per* well.

Cell proliferation after stress was reported as per cent of a normalized value of the same cells counted before exposure to the chemical stressors. Each experimental point was repeated at least three times in duplicate wells, and the analysis made with the combined data.

Data obtained by counting the cells from each condition showing the different patterns of FUS subcellular expression (i.e., nuclear, cytoplasmic, both) were normalized and calculated as per cent of the total number of counted cells. Data were expressed as means ± SD of three separate experiments, each done in duplicate dishes.

We did densitometry analysis of the immunoblot filters with the Image J software.

The proportion of cells containing the rounded foci at different times after stress was expressed as means ± SD. The analysis was made with the combined data of three independent experiments, each done in duplicate dishes.

We used the SIGMASTAT 8.0 software package (Systat Software Inc., San Jose, CA, United States) to perform the statistical analyses. Differences in the proportion of the different patterns of FUS staining and the granule-containing cells were analyzed with One-Way ANOVA, followed by *post hoc* Holm-Sidak analysis. *P*-values < 0.05 were considered significant.

## Results

We used the SH-SY5Y human neuroblastoma cell line, which expresses human FUS (hFUS) carrying a deletion of the NLS domain to study the cell proliferation, the subcellular distribution of the mutant protein and the effect of either acute or chronic stress.

The deletion of this domain mimics the impact of those FUS mutations that impair the nuclear transport of the protein (Dormann et al., 2010; Ederle and Dormann, 2017; Fernandes et al., 2018; Caputo et al., 2020).

### The Cell Proliferation Rate Is Related to the Active Nuclear Transport of Fused-in-Sarcoma Protein Through Its Nuclear Localization Signal

We studied the time course of the SH-SY5Y cells proliferation expressing either homozygous or heterozygous NLS deletion of the FUS protein compared to the WT cells. Twenty-four hours after plating, two of us counted cells at different times, each blinded for the specific cell type counted.

**FIGURE 1:**
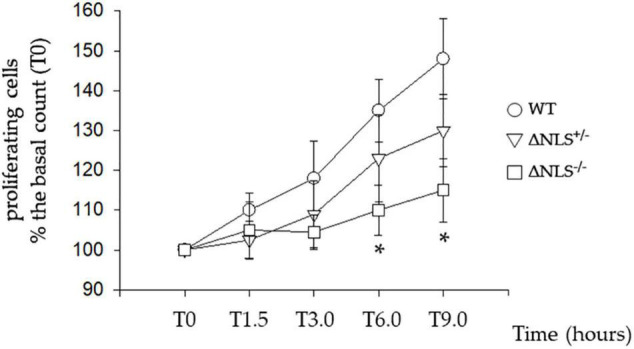
Slow proliferative rate of the SH-SY5Y cells expressing hFUS with a heterozygous (DNLS^+/–^) or a homozygous (DNLS^–/–^) deletion of the Nuclear Localization Signal Domain. Cells were seeded 24 h before the assessment of their proliferation, made by direct counting. Data were normalized to T0, which indicates the time of the first counting after cell plating. Subsequent time points (in hours) for cell counting were T1.5, T3.0, T6.0, and T9.0. Wild Type SH-SY5Y cells; ΔNLS^+/–^ cells; ΔNLS^–/–^ cells. *p < 0.05 ΔNLS^–/–^ cells vs. Wild Type cells; one-way ANOVA with *post-hoc* Holm-Sidak analysis.

Figure 1 shows that the WT cells exhibited a steep growth rate, increasing to 30% from the basal at 6 and 48% at 9 h. The proliferation was slower in the FUS-mutated cell lines; in particular, at 9 h, the ΔNLS^+/–^ cells had increased by 30%, whereas the ΔNLS^–/–^ cells grew only by 15% over the basal. These data, therefore, demonstrate that FUS affects the proliferation rate in the undifferentiated SH-SY5Y cells, which is strongly impaired when the protein lacks its NLS. We observed a similar effect in cultured fibroblasts taken from an asymptomatic carrier of a P525L FUS mutation located in the NLS of the protein (Notaro A, *unpublished observations*).

### Fused-in-Sarcoma Lacking the Nuclear Localization Signal Mislocalizes to the Cytoplasm

**FIGURE 2:**
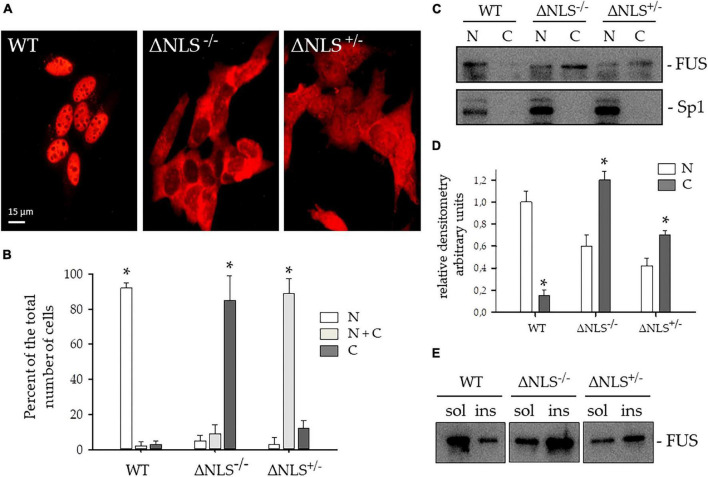
Expression and subcellular distribution of FUS protein lacking the Nuclear Localization (NLS) signal in SH-SY5Y cells. **(A)** Representative immunofluorescence images were performed with the polyclonal anti-hFUS antibody on SH-SY5Y cells, either wild type (WT) or expressing FUS with a deletion of the NLS, either homozygous (ΔNLS^–/–^) or heterozygous (ΔNLS^+/–^). Bar, 10 μm. **(B)** Per cent of the number of cells with FUS expression in the nucleus (N, white column), in the cytoplasm (C, dark gray column), or both (N+C, light gray column) of the WT, ΔNLS^–/–^ and ΔNLS^+/–^ SH-SY5Y cells. Data are expressed as percentages of the total counted cells (mean ± SD of 3 separate experiments, each performed in duplicate wells). *p < 0.05, one-way ANOVA with *post-hoc* Holm-Sidak analysis. **(C)** Western blot of subcellular fractions of WT, ΔNLS^–/–^ and ΔNLS^+/–^ cells stained with a polyclonal anti-hFUS antibody. The polyclonal Sp1 antibody marked the nuclear extract. N, nuclear fraction; C, cytoplasmic fraction. The image is representative of three independent experiments. **(D)** Densitometric analysis of the FUS-related band intensity in the nuclear (N) and cytoplasmic **(C)** fractions. *p < 0.05, nucleus vs. cytoplasm; one-way ANOVA with *post-hoc* Holm-Sidak analysis. **(E)** Western blot of Triton X-100- soluble (sol) and insoluble (ins) fractions of WT, ΔNLS^–/–^ and ΔNLS^+/–^ SH-SY5Y cells stained with a polyclonal anti-hFUS antibody. FUS was over-represented in the insoluble extracts from ΔNLS^–/–^ and ΔNLS^+/–^ cells. The image is representative of three independent experiments.

In both SH-SY5Y ΔNLS^–/–^ and ΔNLS^+/–^ cell lines, FUS heavily mislocalized to the cytoplasm (Figure 2A). In particular, while near one-hundred per cent of the WT cells FUS showed an exclusive nuclear expression, in the vast majority of the ΔNLS^–/–^ cells FUS was present in the cytoplasm, with less than 20% showing a concomitant nuclear expression. Conversely, more than 80% of the heterozygous SH-SY5Y ΔNLS^+/–^ cells displayed a uniform nuclear-cytoplasmic pattern of staining, while in the remaining cells, the mutant protein was expressed only in the cytoplasm (Figure 2B).

This result strongly suggests a dose-effect of the FUS NLS deletion, which fully reproduces the staining pattern seen in fibroblasts with the very aggressive P525L FUS mutation at the C-terminus (Caputo et al., 2020).

The cytoplasmic mislocalization of the NLS-lacking FUS protein was further analyzed in immunoblot. Nuclei were separated from the cytoplasm with the digitonin method (Supplementary Figure 2), and the FUS protein was stained with a polyclonal antibody.

As shown in Figure 2C, WT cells expressed FUS in the nucleus, whereas a FUS-related cytoplasmic band was detectable in homozygous and heterozygous ΔNLS cells. Sp1 antibody allowed identification of the nuclear fraction.

The relative densitometry of the FUS-related bands in the nuclear and cytoplasmic fractions gave a semiquantitative estimation of protein level in the cytoplasm of the two homozygous and heterozygous NLS-deleted cell lines as compared to the WT cells (Figure 2D).

Next, we performed immunoblots of FUS protein in soluble and insoluble cell fractions (Figure 2E). The wild-type FUS was predominantly expressed in the soluble fraction, whereas NLS-deleted FUS increased in the insoluble fraction, indicating its propensity to form aggregates.

### NaAsO_2_–Mediated Acute Stress Induces Stress Granules Formation in Fused-in-Sarcoma Δ Nuclear Localization Signal SH-SY5Y Cells

We performed an acute stress paradigm by exposing the WT and the FUS NLS-deleted SH-SY5Y cells to 0.5 mM NaAsO_2_ for 1 h. Cells were double-stained with anti-FUS and anti-TIA-R antibodies. We also performed double staining with TIA-R and G3BP1 to confirm SGs and found a complete co-localization of the two SGs makers in the small cytoplasmic foci after stress induction (Supplementary Figure 5).

**FIGURE 3:**
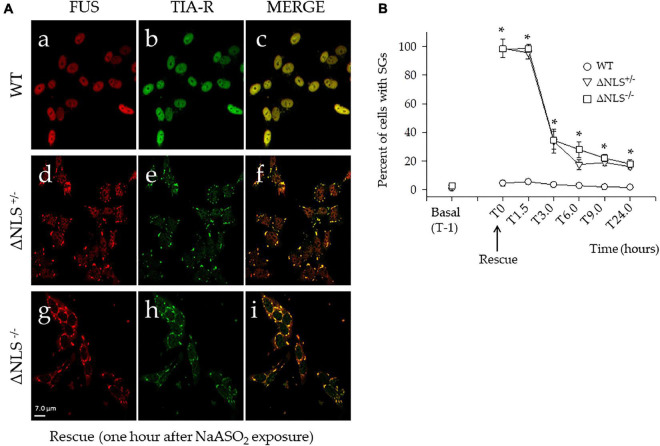
Acute stress with NaASO_2_ induces the appearance of long-lasting stress granules (SGs) in ΔNLS^–/–^ and ΔNLS^+/–^ SH-SY5Y cells, but not in the WT cells. **(A)** Representative confocal images of co-immunofluorescence experiments using antibodies against FUS and TIA-R after acute stress. Cells were fixed and processed at rescue, i.e., 1 h after NaAsO_2_ exposure. While virtually no WT cells showed SGs (panels a–c), the membrane-less organelles were abundant in nearly every cell of the ΔNLS^+/–^ (panels d–f) and ΔNLS^–/–^ (panels g–i) mutants. Scale bar is 10 μm. **(B)** A panel displaying the proportion of cells with SGs at the rescue (T0) and at different time points up to 24 h. The SGs decreased in ΔNLS^–/–^ and ΔNLS^+/–^ cells. However, at 24 h after the rescue, some 20% of the mutant cells still expressed the cytoplasmic organelles. Wild Type SH-SY5Y cells; ΔNLS^+/–^ cells; ΔNLS^–/–^ cells. *p < 0.05, ΔNLS^–/–^ and ΔNLS^+/–^ vs. WT; one-way ANOVA with *post-hoc* Holm-Sidak analysis.

Notably, after stress exposure, only in a few WT cells (i.e., less than 10%) SGs were detected with the two FUS and TIA-R proteins (Figure 3A), being co-localized mainly in the nucleus (Figures 3Aa–c). Conversely, after NaAsO_2_ removal (T0), almost all ΔNLS^–/–^ and ΔNLS^+/–^ cells showed the presence of cytoplasmic stress granules, with a full co-localization of FUS and TIA-R (see Figures 3Ad–f for the ΔNLS^+/–^ cells and Figures 3Ag–i for the ΔNLS^–/–^ cells).

The time-course of the SGs expression (T0–T24 h) in the ΔNLS^+/–^ and ΔNLS^–/–^ cells clearly showed a significant decrease of these structures. At 6 h after the rescue, the number of cells with SGs had decreased to some 20–30%, a value that did not show significant changes up to 24 h (Figure 3B).

We then asked whether the persistent cytoplasmic puncta were the expression of long-lasting SGs-derived protein aggregates. Therefore, the three cell types were incubated with NaAsO_2_ in the presence of 50 μM Cycloheximide (CHX), a well-known SGs inhibitor. Preliminary experiments had shown that CHX was not toxic to the cells (*data not shown*).

**FIGURE 4:**
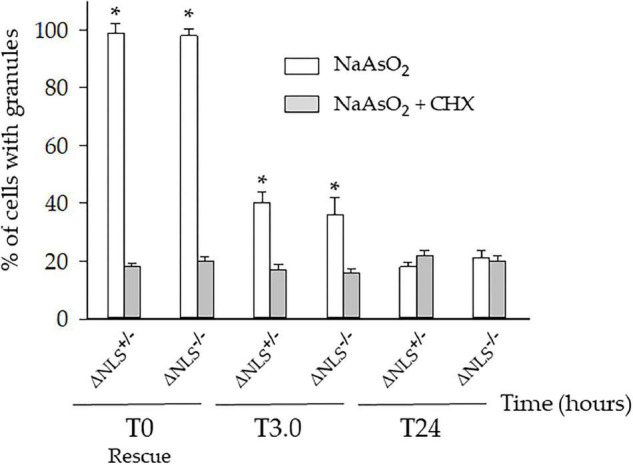
A significant fraction of the NaAsO_2_-induced cytoplasmic organelles persists in ΔNLS^–/–^ and ΔNLS^+/–^ SH-SY5Y cells even after exposure to the SGs inhibitor cycloheximide. FUS ΔNLS^–/–^, FUS ΔNLS^+/–^ and WT cells were co-treated with NaAsO_2_ and cycloheximide for 1 h and then rescued by washing out the chemicals. Cells were double-stained with anti-hFUS and anti-TIA-R antibodies and analyzed with a confocal microscope. The histogram shows the per cent of cells with granules after treatment with NaAsO_2_ alone (white columns) or in combination with cycloheximide (CHX, gray columns). The cells containing the cytoplasmic organelles were counted at the rescue (T0) and after 3 h (T3) and 24 h (T24). Data are expressed as mean ± SD of three independent experiments, each done in duplicate wells. *p < 0.05, NaAsO_2_ vs. NaAsO_2_ + cycloheximide; one-way ANOVA with *post hoc* Holm-Sidak analysis.

One hour after co-incubation of NaAsO_2_ and CHX (i.e., at T0), the number of the ΔNLS^–/–^ and ΔNLS^+/–^ cells showing SGs had significantly decreased, but still, 18–20% of the cells maintained FUS/TIA-R positive granules. This value remained relatively stable at the subsequent time points up to 24 h after rescue (Figure 4).

**FIGURE 5:**
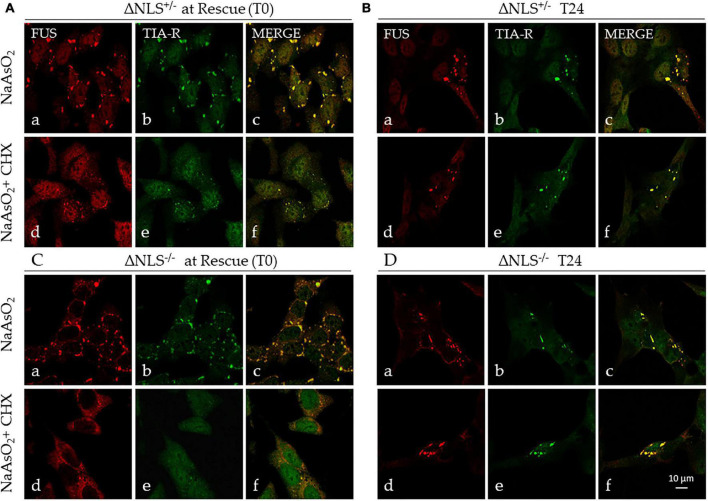
Stress induces persistent cytoplasmic FUS- and TIA-R-positive inclusions. Representative confocal images of FUS and TIA-R double-immunofluorescence in ΔNLS^–/–^ and ΔNLS^+/–^ SH-SY5Y cells after exposure to NaAsO_2_ or NaAsO_2_ + cycloheximide. Analysis was performed at the rescue (T0) and 24 h after rescue (T24). **(A)** FUS ΔNLS^+/–^ cells at T0 after exposure to NaAsO_2_ alone (panels a–c) or to NaAsO_2_ + cycloheximide (panels d–f); **(B)** FUS ΔNLS^+/–^ cells at T24 after rescue (NaAsO_2_ alone: panels a–c; NaAsO_2_ + cycloheximide: panels d–f). **(C)** FUS ΔNLS^–/–^ cells at T0 after exposure to NaAsO_2_ (panels a–c) or to NaAsO_2_ + cycloheximide (panels d–f); **(D)** FUS ΔNLS^+/–^ cells at T24 after rescue (NaAsO_2_ alone: panels a–c; NaAsO_2_ + cycloheximide: panels d–f). Scale bar is 10 μm.

The immunocytochemical analysis confirmed the full co-localization of FUS and TIA-R in these persistent cytoplasmic aggregates (Figure 5). At 24 h, several of SGs lost their puncta characteristics to become larger structures (Figures 5B,D).

We conclude that the CHX-resistant cytoplasmic structures are very likely SG-derived stable insoluble aggregates containing mutant FUS.

The cells expressing FUS with the deletion of the NLS and the WT ones were submitted to chronic stress, using a paradigm of continuous exposure to 10 μM NaAsO_2_ for 24 h.

At the rescue time, all cells, either the WT or those carrying the NLS deletion, were viable but did not show SGs, and they grew normally in culture (*data not shown*). Therefore, the cell responsiveness to chronic stress with arsenite may depend on the specific cell type studied, as human fibroblasts showed to be sensitive to this stress paradigm (Ratti et al., 2020).

## Discussion

This study demonstrates that in a human neuroblastoma SH-SY5Y cell line, engineered to express FUS deprived of the NLS, the protein is heavily misplaced to the cytoplasm where it enters into SGs upon stress.

A significant number of these SGs do not disassemble after the rescue, and they tend to persist in the cells. This data indicates that the FUS-containing inclusions seen in the genetic variants of ALS with FUS mutations at the C-terminus originate from SGs converted from a liquid-liquid to a liquid-solid state.

Our data, therefore, offer important clues to a better understanding of the pathogenesis of Amyotrophic Lateral Sclerosis, and it may pave the way for future personalized therapies.

In the last few years, there has been a wealth of research on SGs formation and dynamics (Anderson and Kedersha, 2008; Khalfallah et al., 2018; Riggs et al., 2020) and their putative role in motor neuron degeneration occurring in ALS (Li et al., 2013; Aulas and Vande Velde, 2015; Ederle and Dormann, 2017; Fernandes et al., 2018; Baradan-Heravi et al., 2020).

Stress granules are membrane-less organelles that contain mRNA, RNA-binding proteins (RBP) and other proteins. They form upon cell exposure to a stressful agent, which could be infectious (e.g., a virus), physical (e.g., heat-shock, osmotic shock, UV, hypoxia) or chemical (e.g., dithiothreitol, paraquat, sodium arsenite).

Stress induces polysome disassembly and blocks protein synthesis. Consequently, mRNA is stalled in translation, trapped along with the RBPs in the SGs organelles until recovery (Anderson and Kedersha, 2008; Hofmann et al., 2021). Therefore, stress granules help the cell cope with external stress by reducing the protein synthesis and, therefore, the cell metabolism and activity. Besides the coalescence of RNA and proteins in such granules, these organelles remain in a liquid state. After stress removal, in physiological conditions SGs disassemble, allowing the recovery process (Hofmann et al., 2021). In pathological conditions, like the neurodegenerative process in ALS, the disassembling process might become impaired (Buratti and Barrale, 2010; Li et al., 2013; Fernandes et al., 2018). This process most probably occurs because of a higher protein density in the granules, with a consequent liquid-to-solid phase change of SGs, exceedingly facilitated by ALS-related proteins with a prion-like low-complexity domain, like TDP-43, Ataxin-1, Ataxin-2 and FUS (Li et al., 2013; Pakravan et al., 2021). In particular, the RNP FUS is a critical component of SGs after stress (Dormann et al., 2010; Lo Bello et al., 2017). A wealth of evidence shows that increasing cytoplasmic concentration of FUS or mutations in the prion-like domain at the N-terminus promotes the protein’s conversion into an aggregated state (Shelkovnikova et al., 2014; Patel et al., 2015). Conversely, the interaction of the NLS-PY domain with either TNPO-1 or with the G quadruplex sequences on mRNAs bound by the protein facilitate maintaining a liquid-liquid state (Yoshizawa et al., 2018; Ishiguro et al., 2021). Mutations at the NLS-PY domain, e.g., the P525L, revert this condition by inducing a liquid-to-solid transition (i.e., an aggregated state).

Therefore, those NLS-PY mutations leading to a high cytoplasmic level of FUS may facilitate a liquid-to-solid phase transition of FUS-containing SGs, allowing the formation of cell-insoluble inclusions and the consequent cell damage and death.

Mutations of the FUS protein at the NLS domain cause a form of ALS with an aggressive phenotype (Chiò et al., 2009; Conte et al., 2012; Kuang et al., 2017), being also responsible for the massive nucleus-to-cytoplasm misplacement of the protein (Kwiatkowski et al., 2009; Vance et al., 2009, 2013; Dormann et al., 2010; Lo Bello et al., 2017). As pointed, the mutant FUS mislocalized to the cytoplasm incorporates into SGs upon stress exposure (Dormann et al., 2010; Li et al., 2013; Ederle and Dormann, 2017; Lo Bello et al., 2017; Dudman and Qi, 2020). Whether the FUS-containing SGs effectively coalesce into insoluble cytoplasmic inclusions has been a matter of debate (Buratti and Barrale, 2010; Aulas and Vande Velde, 2015). However, there is evidence of the presence of stress proteins into the cytoplasmic inclusions in motor neurons of ALS patients with FUS mutations (Dormann et al., 2010). We recently demonstrated that in fibroblasts taken from asymptomatic FUS P525L mutation carriers, FUS misplaces the cytoplasm and transiently incorporates into SGs upon acute stress. Acute stress caused the formation of FUS-containing SGs also in fibroblasts from sporadic ALS, with no known mutations. Still, they dissolved earlier than those formed in fibroblasts of FUS P525L mutation carriers, suggesting in the latter a pre-aggregative state (Lo Bello et al., 2017).

Why did the SGs in the mutant fibroblasts not convert to permanent inclusions? One possibility might be that fibroblasts are unsuitable for forming pathological marks of ALS or that the amount of mutant FUS protein misplaced to the cytoplasm was not high enough to force a liquid-to-solid phase transition of SGs to stable inclusions upon stress. Alternatively, prolonged (chronic) stress might be necessary to induce the formation of stable aggregates into the cells, as it was recently demonstrated in fibroblasts and iPSC-derived motor neurons carrying a TARDBP pA382T mutation or C9 or f72 pathological expansions (Ratti et al., 2020).

To answer these questions, we used the undifferentiated SH-SY5Y cells engineered to express NLS-deleted FUS protein, either as heterozygous or homozygous (An et al., 2019).

There were already essential differences between the mutant FUS ΔNLS cells and the WT cells in basal conditions. The mutant cells did not grow as fast as the WT, and FUS was heavily misplaced to the cytoplasm. Furthermore, the cell growth was particularly slow in the homozygous ΔNLS^–/–^ cells, suggesting that the displacement of mutant FUS in the cytoplasm has a deleterious effect on proliferation. The cytoplasmic FUS displacement and slow cell proliferation were already observed in fibroblasts carrying FUS P525L mutation (Lo Bello et al., 2017).

A possible explanation of this effect might be either altering the mRNA activity in the cytoplasm or modifying the pre-mRNA transcription and processing into the nucleus (Ederle and Dormann, 2017).

The presence of mutant FUS in the cytoplasm is a feature of the mutations involving the C-terminal, or when deletion of the NLS domain is performed, similar to the present study (Kwiatkowski et al., 2009; Vance et al., 2009, 2013; Dormann et al., 2010; Kuang et al., 2017; Lo Bello et al., 2017). However, recent studies suggest that cytoplasmic FUS can also be a hallmark of sporadic ALS or the disease due to other genetic mutations, as VCP (Tyzack et al., 2019).

In our model, a uniform FUS misplacement to the cytoplasm was observed in both ΔNLS^+/–^ and ΔNLS^–/–^ cells but with a striking difference in the nuclear protein expression. FUS was almost absent in the nucleus of nearly all homozygous ΔNLS cells, which is an abnormal distribution for this nuclear RBP. Note that a similar phenomenon was seen in degenerating motor neurons of transgenic mice with a conditional expression of hFUS P525L and in fibroblasts of FUS P525L mutation carriers soon after phenoconversion, wherein 2/3 of the mutant protein cells were not expressed in the nucleus (Sharma et al., 2016; Caputo et al., 2020). Interestingly, no cytoplasmic aggregates were detected in the cytoplasm of both the transgenic mouse and the P525L mutant fibroblasts.

Other FUS mutations in the NLS-PY domain do not show the peculiar expression of the P525L mutation, with a proportion of the protein present in the cytoplasm generally higher than in the nucleus. For instance, the fibroblasts carrying the quasi-identical P525R mutation expressed the mutant FUS prominently in the nucleus, with a relatively minor presence in the cytoplasm (Kuang et al., 2017). These data strongly suggest that the proline-to-leucine amino acid change at position 525 of FUS protein hampers its ability to be imported to the nucleus much more than other mutations at the C-terminus. Yet, both P525L and P525R mutations lead to an aggressive form and juvenile-onset ALS (Chiò et al., 2009; Conte et al., 2012; Kuang et al., 2017).

The P525L mutation, however, seems to reflect better the deletion of the NLS domain, which prevents the protein from binding mainly to TNPO-1 with consequent reduction of its import into the nucleus (Dormann et al., 2010, 2012; Baade et al., 2021).

The resulting elevated cytoplasmic FUS protein levels in cells with the P525L mutation or with the NLS deletion represent the basis for its strong propensity to enter into SGs massively, thus possibly facilitating the formation of permanent, SGs–derived, cytoplasmic inclusions (Ederle and Dormann, 2017; Dudman and Qi, 2020; Pakravan et al., 2021). FUS-containing inclusions have been observed in sporadic and familial ALS (Deng et al., 2010; Tyzack et al., 2019) and mice overexpressing the wild type protein (Mitchell et al., 2013).

The acutely induced stress with 0.5 mM NaAsO_2_ did not produce significant FUS-containing SGs in the WT cells (less than 5% of them showed SGs), whilst almost the totality of the mutant cells, either ΔNLS^–/–^ or ΔNLS^+/–^, displayed abundant cytoplasmic FUS- and TIA-R-positive foci. This latter data was expected, as the mutant proteins, FUS P525L and FUS P525R, entered massively into SGs of mutant fibroblasts and transfected HeLa cells (Dormann et al., 2010; Kuang et al., 2017; Lo Bello et al., 2017).

The evidence that only a minority of the WT SH-SY5Y cells produced SGs after exposure to arsenite suggests that these cells may be more resistant than other cells types to this paradigm of oxidative stress.

The time course of the SGs after rescue in our model confirmed the notable decrease of the membrane-less organelles already at 3 h of recovery (Lo Bello et al., 2017). Still, it demonstrated their significant persistence at the following time points. Similar long-lasting SGs, which therefore apparently lost their physiological property to assemble and then to disassemble (Riggs et al., 2020; Hofmann et al., 2021), were seen in fibroblasts carrying either a TDP-43 A382T mutation or a C9 or f72 pathological expansion (Ratti et al., 2020).

In our model, exposure to cycloheximide (CHX), a molecule able to prevent the SGs formation, strongly decreased but did not abolish the presence of FUS- and TIA-R-positive foci in FUS ΔNLS-expressing cells up to 24 h after rescue. Even more intriguing, CHX-resistant granules were present already at the rescue. This data suggests that the FUS/TIA-R-positive SGs have switched from a liquid-to-solid phase, evolving toward stable SG-derived inclusions (Siwach and Kaganovich, 2017).

Therefore, this is the first direct evidence that FUS-containing inclusions seen in sporadic and genetic ALS might derive from long-lasting SGs (Deng et al., 2010; Ederle and Dormann, 2017; Tyzack et al., 2019). It also implies a putative involvement of endogenous or exogenous stress in the pathophysiology of ALS.

One important exogenous stress-inducer in ALS might be a virus infection. Recent work has shown that the human endogenous retrovirus-K (HERV-k) is activated in ALS through its interaction with TDP-43 (Li et al., 2015). The HERV-k RNA transport factor Rec protein forms a complex with Staufen-1 facilitating the TDP-43 mislocalization to the cytoplasm. Both Rec-Staufen complex and TDP-43 enter into SGs upon stress (Hanke et al., 2013). Furthermore, infection with Theiler’s murine encephalomyelitis virus in cell models triggers TDP-43 mislocalization and incorporation into SGs (Masaki et al., 2019). In addition, an antiviral immune response to a virus infection/activation promotes persistent mutant FUS accumulation into SGs-derived inclusions (Shelkovnikova et al., 2019).

These studies suggest that viruses and the antiviral immune response might be important players for ALS-related proteins mislocalization and incorporation into persistent aggresomes, contributing to ALS proteinopathy.

**FIGURE 6:**
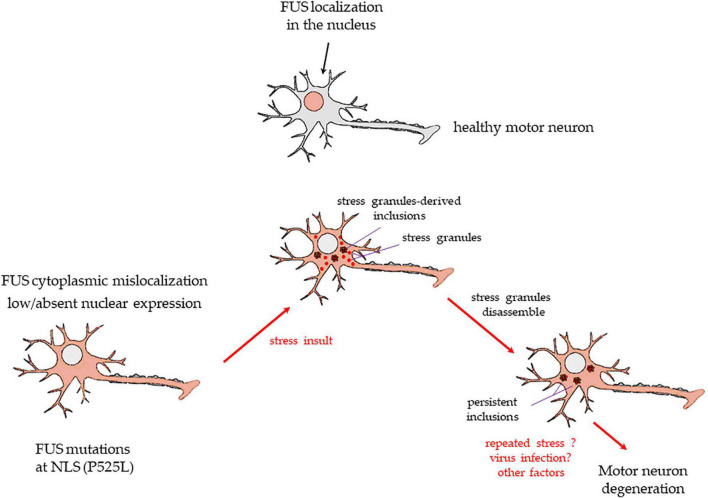
A drawing showing the putative mechanism of transformation of SGs into persistent cytoplasmic inclusions in motor neurons, based on the present study results. Upper image: a healthy motor neuron with FUS expressed in the nucleus. FUS mutations at the C-terminus, e.g., P525L, or deletions of the NSL domain, as in the present study, cause a massive FUS mislocalization to the cytoplasm and low/absent nuclear levels of the protein (lower image on the left). A stressful event (physical? infective? inflammatory? other causes?) induces healthy motor neurons to form membrane-less cytoplasmic SGs. Usually, SGs are in a liquid-liquid phase, and they dissolve readily after stress termination. However, in cells with FUS mutations/deletions at the NLS domain, the protein enters massively in the SGs. A number of these organelles may change to liquid-to-solid phase and coalesce into persistent inclusions (lower image in the middle). After the stress is terminated, SGs readily dissolve, but inclusions do not (lower image on the right). Repeated stress events or other factors (e.g., virus infection, antiviral immune response) may lead to further FUS-containing insoluble inclusions. The resulting alterations of FUS-related mRNA activity and transport in the motor neurons may strongly contribute to the neurodegenerative process seen in ALS patients bearing the FUS mutations at the C-terminus.

Based on our experiments, Figure 6 shows a proposed mechanism in which stress may induce in motor neurons carrying FUS mutations at the C-terminus, the appearance of both SGs and inclusions. While SGs disassemble, inclusions persist, and with the contribution of other pathogenic factors (e.g., repeated stress exposure, virus infection), they may become permanent, contributing to cell degeneration and death.

The prolonged 24 h exposure to relatively low, non-toxic doses of arsenite was ineffective in our model. Neither the WT nor the mutant FUS ΔNLS cells showed SGs at rescue, and the cells proliferated normally in culture. Loss of arsenite’s efficacy is unlikely; as in other cell models, prolonged exposure to low doses of arsenite did induce SGs formation (Ratti et al., 2020). It, therefore, reinforces the hypothesis that the SH-SY5Y cell line is relatively resistant to stress.

## Conclusion

In conclusion, our experiments show that it is possible to induce, through acute stress, persistent SGs in the SH-SY5Y cell model overexpressing hFUS with a deletion of its NLS. The effect appears quite strong in the homozygous cells where FUS was heavily misplaced to the cytoplasm and absent into the nucleus. Therefore, the persistent FUS-containing SGs might evolve to form stable inclusions, a hallmark of ALS and Frontotemporal dementia, either sporadic or genetic (Deng et al., 2010; Amstrong, 2017; Tyzack et al., 2019).

FUS binds tightly to mRNA in the nucleus, and it goes back to the cytoplasm through a mechanism independent of an Exportin1/CRM1 interaction (Ederle and Dormann, 2017; Ederle et al., 2018). Therefore, FUS with mutations at the C-terminus not only is misplaced to the cytoplasm because of a compromised nuclear transport but its retention to the nucleus is reduced by passive diffusion (Dormann et al., 2010; Ederle et al., 2018). The nucleus becomes progressive empty of FUS, and the protein heavily accumulates in the cytoplasm (Sharma et al., 2016; Tyzack et al., 2019; Caputo et al., 2020).

This mechanism, along with the prion-like properties of FUS, makes the protein prone to form inclusions and to massively enter the SGs, where it probably contributes to the liquid-to-solid phase transition of the membrane-less organelles that then may coalesce into insoluble aggregates (Patel et al., 2015; Siwach and Kaganovich, 2017).

Our work adds to the knowledge on the pathogenesis of FUS-containing inclusions. However, further studies are needed to understand better this mechanism and its putative implication in ALS pathophysiology.

## Data Availability Statement

The original contributions presented in the study are included in the article/Supplementary Material, further inquiries can be directed to the corresponding author/s.

## Author Contributions

AN performed the experiments, analyzed the data, and wrote the first draft of the manuscript with all subsequent revisions. AM performed the experiments and contributed to the data analysis and to the manuscript revisions. VL designed the study, analyzed the data, supervised the statistical analysis, and wrote the first draft of the manuscript and all subsequent revisions. All authors contributed to the article and approved the submitted version.

## Conflict of Interest

The authors declare that the research was conducted in the absence of any commercial or financial relationships that could be construed as a potential conflict of interest.

## Publisher’s Note

All claims expressed in this article are solely those of the authors and do not necessarily represent those of their affiliated organizations, or those of the publisher, the editors and the reviewers. Any product that may be evaluated in this article, or claim that may be made by its manufacturer, is not guaranteed or endorsed by the publisher.

## Abbreviations

ALS: Amyotrophic Lateral Sclerosis
FUS: Fused-in-Sarcoma
NLS: Nuclear Localization Signal
SG: Stress Granules
TIA-R: T-cell intracellular antigen 1-related protein
G3BP1: GTPase-activating protein-binding protein 1
WT: Wild-Type
NaASO_2_: Sodium Arsenite
CHX: Cycloheximide
MN: Motor Neurons
RNP: RNA/DNA binding protein.
