# Supplementary figures and images for: A Deletion of the Nuclear Localization Signal Domain in the Fus Protein Induces Stable Post-stress Cytoplasmic Inclusions in SH-SY5Y Cells
Source: Front Neurosci. 2021 Dec 23;15:759659. doi: 10.3389/fnins.2021.759659 (PMC8733393; doi:10.3389/fnins.2021.759659)

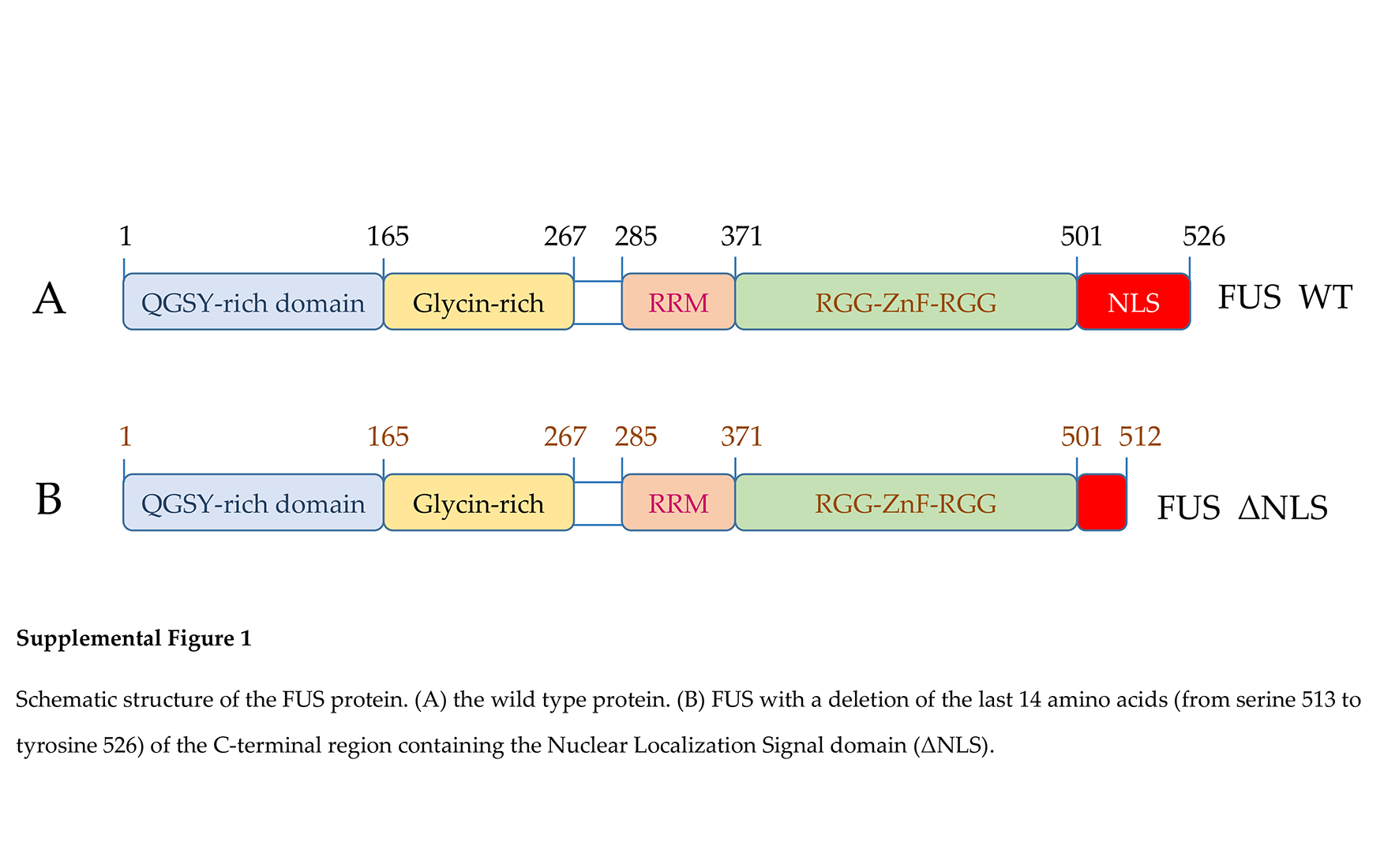

Supplement: Supplementary file 1 [file Image_1.tif]

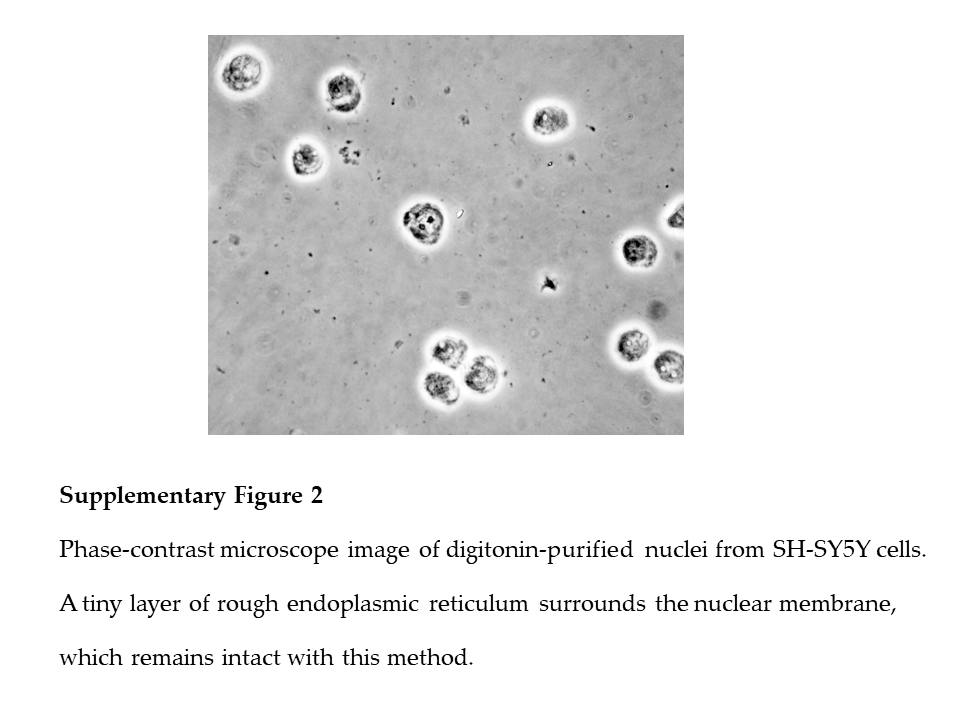

Supplement: Supplementary file 2 [file Image_2.tif]

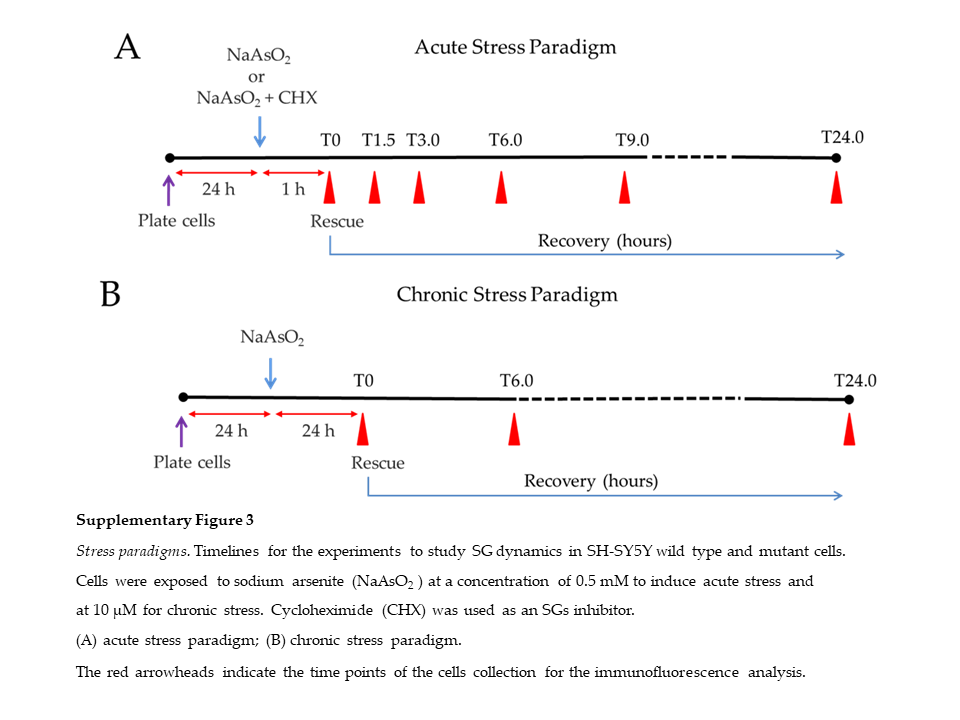

Supplement: Supplementary file 3 [file Image_3.tif]

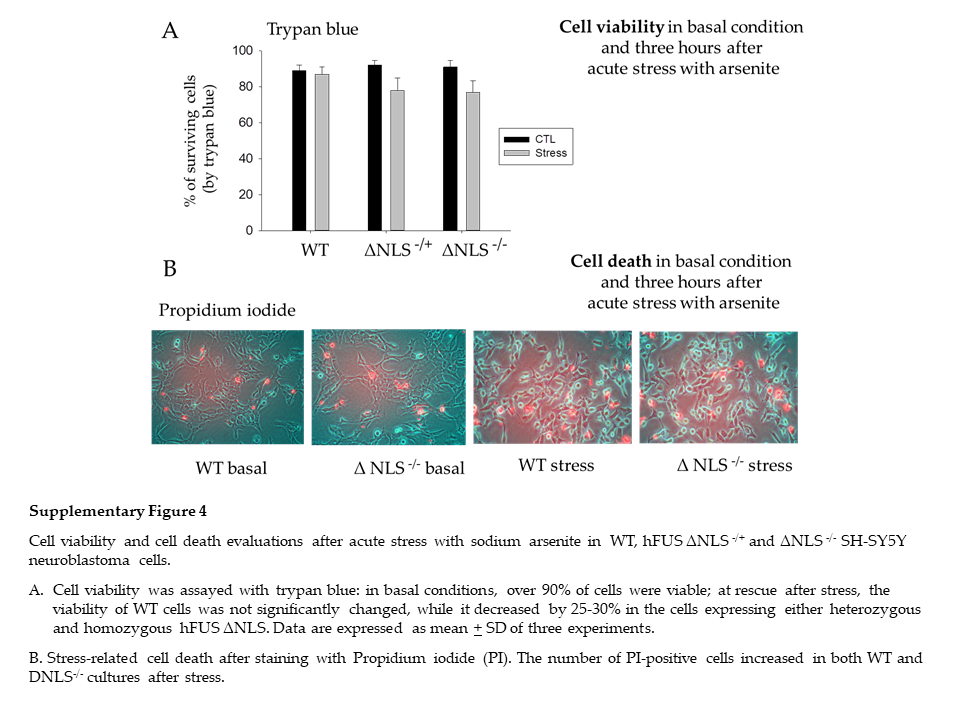

Supplement: Supplementary file 4 [file Image_4.tif]

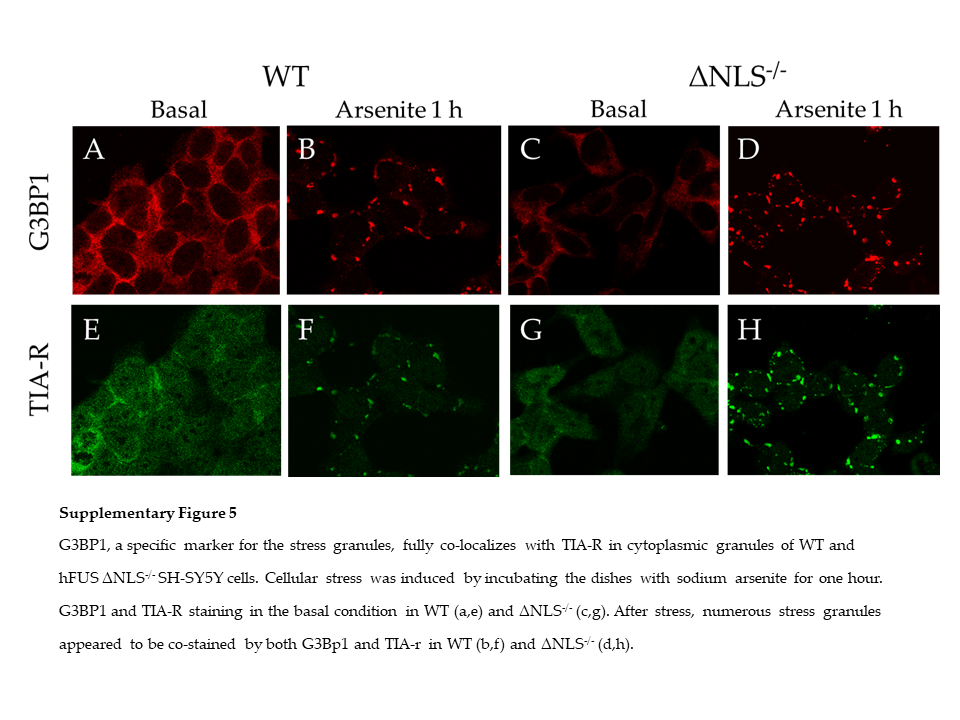

Supplement: Supplementary file 5 [file Image_5.tif]
